# Biocircular economy-driven bacterial cellulose with large pore size: statistical optimization using *Glutamicibacter soli* bread waste hydrolysate

**DOI:** 10.1186/s40643-026-01098-1

**Published:** 2026-07-27

**Authors:** Fatma Abdul EL Hak, Amira A. Matrawy, Mohamed S. Elnouby, Amira M. Embaby, Wael Wazeer, Hoda E. Mahmoud

**Affiliations:** 1 Biotechnology Department, Institute of Graduate Studies and Research, Alexandria University, Alexandria, Egypt; 2 Environmental Studies Department, Institute of Graduate Studies and Research, Alexandria University, Alexandria, Egypt; 3 Nanomaterials and Composites Research Department, Advanced Technology and New Materials Research Institute, City of Scientific Research and Technological Applications (SRTA-City), New Borg El-Arab City, Alexandria, Egypt; 4 Electronic Materials Department, Advanced Technology and New Materials Research Institute, City of Scientific Research and Technological Applications (SRTA-City), New Borg El-Arab City, Alexandria, Egypt

**Keywords:** *Komagataeibacter* sp., Bacterial cellulose, Bread waste hydrolysate, Response surface methodology, BC porosity, Waste valorization

## Abstract

Bacterial cellulose (BC) is a versatile biopolymer of considerable biotechnological interest, with wide-ranging applications in biomedical, industrial, and engineering fields attributable to its exceptional structural and physicochemical properties. Despite its promise, the high cost of conventional production media and the challenge of tailoring BC structural characteristics remain critical bottlenecks limiting sustainable large-scale manufacturing. In the present study, a novel fermentation medium based on *Glutamicibacter soli* bread waste hydrolysate (BWH) was developed and statistically optimized for high-yield BC production by *Komagataeibacter* sp. strain HIJ12 EMCCN-4085, integrating circular bioeconomy principles through food waste valorization. One-variable-at-a-time (OVAT) screening identified glycerol and ethanol supplementation, incubation at 28 °C, pH 5.5, a 2-day-old inoculum, and an aeration ratio of zbroth: flask volume) as the key determinants governing BC biosynthesis. Subsequent Box–Behnken design-based Response Surface Methodology, supported by canonical and ridge analyses, refined glycerol concentration, inoculum size, and incubation time, yielding an exceptional BC dry weight of 39 g L^−1^ within 5.8 days, substantially surpassing most reported waste-based BC systems that typically achieve 4–15 g L^−1^ over longer fermentation periods. Structural characterization demonstrated that hydrolysate-based BC possessed a distinctively large pore size, enhanced porosity (41 ± 1.2%), and reduced crystallinity relative to conventional Hestrin–Schramm-based BC, while fully preserving the characteristic cellulose chemical structure, properties particularly advantageous for drug delivery, wound healing, and filtration applications. Collectively, these findings establish *G. soli* BWH as a high-performance, sustainable fermentation substrate that simultaneously enhances BC productivity and tailors material properties, offering a compelling strategy for converting food waste into high-value biomaterials.

**Graphical abstract:**

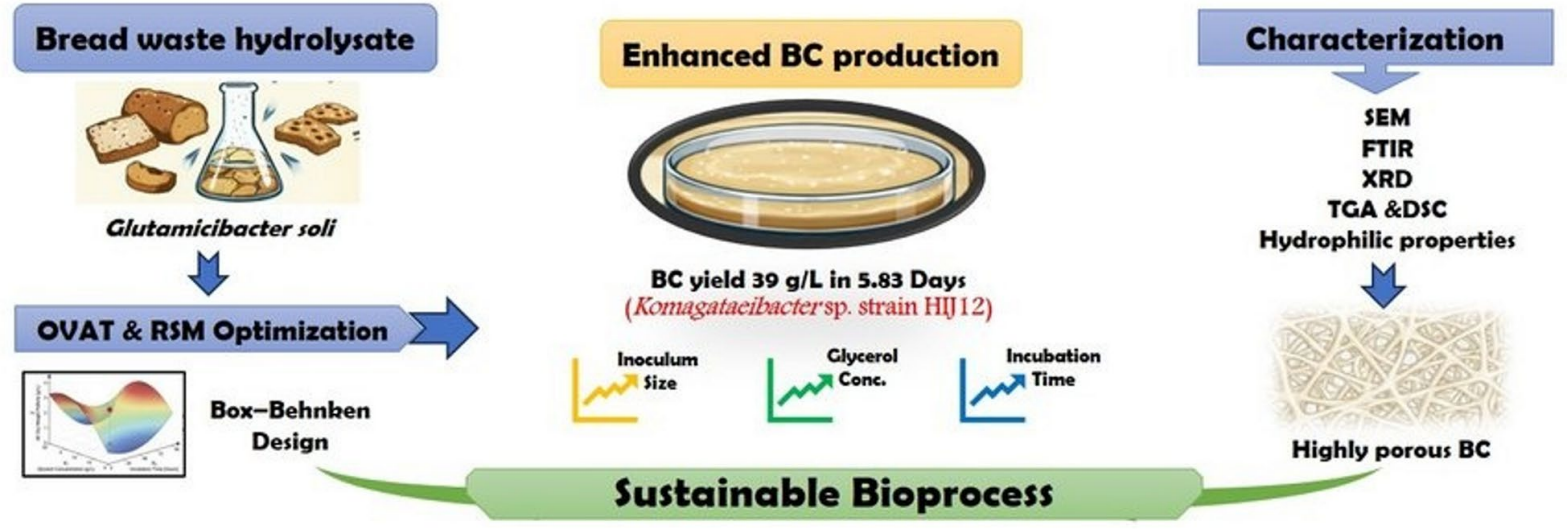

**Supplementary Information:**

The online version contains supplementary material available at 10.1186/s40643-026-01098-1.

## Key points

- *G. soli* bread waste hydrolysate established as a biocircular BC production substrate.
- Box–Behnken RSM with ridge analysis achieved exceptional BC yield of 39 g L^−1^.
- Optimized yield represents a 10-fold enhancement over the unoptimized baseline.
- BC production achieved within 5.8 days, outperforming conventional HS medium.
- Hydrolysate-based BC exhibited larger pore size and enhanced porosity (41%).
- Reduced crystallinity (54%) preserved cellulose I structure for biomedical applications.

**Supplementary Information:**

The online version contains supplementary material available at 10.1186/s40643-026-01098-1.

## Introduction

Bacterial cellulose (BC) has emerged as a structurally superior alternative to plant-derived cellulose, sharing the same chemical composition yet requiring no delignification or hemicellulose removal, and exhibiting exceptional purity, high water-holding capacity, nanofibrillar architecture, biodegradability, biocompatibility, and remarkable mechanical strength in the wet state (Lahiri et al. 2021; Gallegos et al. 2016). These distinctive properties underpin its growing use across diverse high-value sectors, including wound dressings, tissue engineering scaffolds, drug delivery matrices, filtration membranes, and sustainable packaging composites (Gallegos et al. 2016; Eskilson et al. 2024; Klemm et al. 2001; Iqbal et al. 2014). Nevertheless, two interrelated challenges continue to constrain the broader industrial adoption of BC: the high cost of conventional defined media and the limited ability to reproducibly tailor BC structural features, particularly porosity and crystallinity, to match application-specific requirements (Hussain et al. 2019).

Among BC-producing microorganisms, *Komagataeibacter* spp. stand out as the most efficient producers and have been extensively studied across a wide range of substrates and culture strategies (Wang et al. 2018; Jonas and Farah 1998; Skaradziński et al. 2025). Efforts to improve BC productivity have pursued multiple complementary routes, encompassing high-yielding strain isolation, metabolic and genetic engineering to enhance cellulose biosynthesis flux, and systematic optimization of physicochemical culture conditions (Yang et al. 2013; Krystynowicz et al. 2002). At the medium level, carbon and nitrogen source selection exerts a strong influence on both BC yield and material properties; accordingly, diverse substrates, ranging from fructose, sucrose, and glycerol to molasses and various agro-industrial residues, have been evaluated, alongside nitrogen sources such as yeast extract, peptone, and corn steep liquor (Buldum et al. 2017; Velásquez-Riaño and Bojacá 2017; Huang et al. 2016; Carreira et al. 2011). Despite these advances, production cost remains a primary bottleneck, and the rational connection between medium composition and BC structural output remains incompletely understood (Hussain et al. 2019).

The valorization of food waste within a circular bioeconomy framework represents a strategically compelling approach to simultaneously address both challenges, reducing feedstock cost and generating structurally distinct BC (Gallegos et al. 2016). Bread waste is particularly attractive in this context owing to its abundance, high carbohydrate content, and nutrient richness; however, its effective utilization as a fermentation substrate requires conversion into a fermentation-ready hydrolysate containing assimilable sugars and growth-supporting nutrients. While chemical and enzymatic hydrolysis routes have been explored for this purpose, microbial hydrolysis, wherein an amylolytic microorganism directly converts starch-rich substrates into a nutrient-balanced hydrolysate, offers a simpler, more scalable, and potentially more cost-effective alternative that has received comparatively little attention in the BC literature.

*Komagataeibacter* spp. have been successfully cultivated on a variety of low-cost waste-derived substrates, including fermented rice noodle wastewater, vinegar residue hydrolysate, corncob, sugarcane bagasse enzymatic hydrolysate, and bread waste enzymatic hydrolysate (Sutthiphatkul et al. 2020; Zhang et al. 2025; Akintunde et al. 2022; Pilafidis et al. 2025), demonstrating the genus’s metabolic versatility and adaptability to heterogeneous carbon sources. However, the use of microbially generated BWH, particularly those derived from amylolytic bacteria, as BC production media has not been reported, representing a clear gap in the current literature.

Building on our previous work demonstrating optimized α-amylase production by the psychrotolerant *Glutamicibacter soli* strain AM6 EMCCN3074 on bread waste (Matrawy et al. 2023), we recognized that its cell-free culture supernatant is inherently enriched in reducing sugars and oligosaccharides alongside growth-supporting nutrients, a composition that could directly sustain BC biosynthesis by *Komagataeibacter* sp. without further chemical or enzymatic supplementation. Accordingly, this study aimed to: (i) evaluate *G. soli* AM6 EMCCN3074 BWH as a novel, sustainable BC production medium; (ii) apply a two-stage statistical optimization strategy, comprising OVAT screening followed by Box–Behnken design-based Response Surface Methodology with canonical and ridge analyses, to maximize BC yield; and (iii) characterize the structural, thermal, mechanical, and hydrophilic properties of the resulting hydrolysate-based BC in comparison with BC produced on conventional Hestrin–Schramm medium, with particular emphasis on porosity and pore size as application-relevant structural features (Wang et al. 2018; Jonas and Farah 1998; Skaradziński et al. 2025). This integrated approach aligns food waste valorization with precision fermentation optimization, contributing to the growing body of evidence supporting circular bioeconomy strategies for the sustainable production of high-value biomaterials (Montenegro-Silva et al. 2024; Gomes et al. 2013; Jang et al. 2325; Shigematsu et al. 2005).

## Methods

### Microorganisms and cultivation conditions

The cellulose-producing bacterial strain used in this study was isolated from kombucha tea and employed as the starter culture according to a previously reported method (Jayabalan et al. 2007). The isolate was cultivated under static conditions at 28 °C and pH 5.5 for bacterial cellulose (BC) production.

*Glutamicibacter soli* strain AM6 EMCCN3074 and *Penicillium chrysogenum* strain A3 DSM 105774, previously isolated and identified (Matrawy et al. 2023, 2021), were used for the preparation of BWH. In addition, *Aspergillus oryzae* was obtained from the Culture Collection of the Microbial Biotechnology Laboratory, Biotechnology Department, Institute of Graduate Studies and Research, Alexandria University, and was also used during the hydrolysate preparation process.

For cultivation, *G. soli* strain AM6 EMCCN3074 was grown at 20 °C, pH 7.5, with agitation at 150 rpm. These conditions were maintained to obtain the culture supernatant used for BWH preparation.

### Bacteriological media

Hestrin-Schramm (HS) medium (Schramm and Hestrin 1954) was used for activating the bacterial cellulose (BC)-producing strain and served as the control medium for BC production. The HS medium contained (g L^−1^): glucose, 20; peptone, 5; yeast extract, 5; citric acid, 1.15; and Na_2_HPO_4_·12H_2_O, 2.7.

Luria–Bertani (LB) broth was used for the activation and seed culture preparation of *Glutamicibacter soli* strain AM6. The composition of LB broth was (g L^−1^): tryptone, 10; yeast extract, 5; and NaCl, 5.

Potato dextrose agar (PDA), purchased from HiMedia Co. (India), was used for the activation of the fungal strains and for the preparation of fungal spore suspensions used as seed cultures.

### Isolation of BC-producing bacterium

The BC-producing bacterial strain was isolated from kombucha tea using HS medium as the isolation medium, following a previously reported procedure (Jayabalan et al. 2007).

### Evaluation of BC production capability

The ability of the isolated bacterial strain to produce BC was assessed in HS broth. Briefly, single, freshly prepared colonies from HS agar were used to inoculate 20 mL of HS broth in 100 mL Erlenmeyer flasks. The inoculated broth was incubated at 28 °C with agitation at 150 rpm for 48 h. Subsequently, 3 mL of this seed culture was transferred to 30 mL of HS broth in 250 mL Erlenmeyer flasks and incubated under static conditions at 28 °C for 5–6 days. At the end of the incubation period, the synthesized BC pellicles were harvested, allowed to dry, and their weight was measured.

### Identification of the BC-producing bacterium

The bacterial strain was identified via 16S rRNA gene sequencing, according to a previously reported protocol (Eden et al. 1991a). The full-length 16S rRNA gene was PCR-amplified using the universal primer pair 27F (5′-AGAGTTTGATCCTGGCTCAG-3′) and 1492R (5′-GGTTACCTTGTTACGACTT-3′) (Eden et al. 1991b). PCR was performed as described previously (Matrawy et al. 2024), and the PCR products were purified using the GeneJET PCR Purification Kit (Thermo Fisher Scientific, Waltham, MA, USA) following the manufacturer’s instructions. The purified PCR product was sequenced using ABI PRISM BigDye™ Terminator Cycle kits.

The resulting 16S rRNA nucleotide sequence was analyzed using BLASTN 2.17.0 + against the NCBI rRNA/ITS databases (Altschul et al. 1997). A phylogenetic tree was constructed using MEGA version 11.0 to determine the evolutionary relationship of the isolate with closely related bacterial sequences. The 16S rRNA sequence of the BC-producing bacterium was deposited in GenBank under accession number PQ287240.1. The strain itself was deposited in a public culture collection under accession number EMCCN-4085 to ensure accessibility for the scientific community.

### Preparation of bread waste hydrolysates (BWHs)

Three types of BWHs were prepared in this study using *Glutamicibacter soli* strain AM6 EMCCN3074, *Penicillium chrysogenum* strain A3 DSM 105774, and *Aspergillus oryzae*. The hydrolysate production medium contained the following components (% w/v): KH_2_PO_4_, 0.1; Na_2_HPO_4_, 0.2; NaCl, 0.1; (NH_4_)_2_SO_4_, 0.098; MgSO_4_·7H_2_O, 0.005; CaCl_2_, 0.005; and chopped bread waste, 3.8 (Matrawy et al. 2023).

A 24 h seed culture of *G. soli* strain AM6 EMCCN3074 was prepared in LB broth as described above. Fungal spores of each fungal strain (*P. chrysogenum* A3 DSM 105774 and *A. oryzae*) were prepared on potato dextrose agar for 5 days.

Cultivation conditions for hydrolysate production were as follows: *G. soli* strain AM6 EMCCN3074 was incubated at 20 °C, pH 7.5, for 2.4 days, while *P. chrysogenum* strain A3 and *A. oryzae* were incubated at 30 °C, pH 5.5, for 5 days.

At the end of incubation, cultures were centrifuged at 7000 rpm for 5 min to remove bacterial cells, fungal mycelia, or spores. The resulting cell-free supernatants, referred to as BWHs, were stored at − 20 °C until further use.

### Preliminary production of BC using BWH

A seed culture of the isolated BC-producing bacterial strain was prepared by inoculating 20 mL of HS broth in a 100 mL Erlenmeyer flask with single, freshly prepared colonies from HS agar. The inoculated broth was incubated at 28 °C with agitation at 150 rpm for 2 days.

Subsequently, 3 mL of the seed culture was used to inoculate the BC production medium, which consisted of 160 mL of BWH supplemented with 0.8 g yeast extract per 1 L Erlenmeyer flask. The inoculated culture was incubated under static conditions at 28 °C for 10 days to allow BC biosynthesis.

### Statistical optimized BC production

The production of BC by the investigated bacterial strain was subjected to a two-step statistical empirical approach using OVAT (one variable at a time) approach and response surface methodology consecutively.

#### OVAT approach

In OVAT, the effect of the nine physicochemical parameters on BC production was unveiled. The nine physicochemical parameters were different BWHs (*G. soli* BWH, *P. chrysogenum* BWH, and *A. or*yzae BWH), different carbon sources (peanut, sesame, and glycerol), ethanol, different organic nitrogen sources (peptone, casein, tryptone, and beef extract), different inorganic carbon sources (ammonium sulphate, ammonium hydrogen citrate, ammonium dihydrogen orthophosphate, ammonium chloride, and potassium nitrate), inoculum age, incubation temperature, different aeration ratios of broth volume: flask volume, and pH of the production medium. For different carbon sources, the applied concentration for each tested carbon source was 3% (w/v) for peanut and sesame each and 2.5% (v/v) glycerol. However, the applied concentration of each nitrogen source whether inorganic or organic nitrogen source was 0.5% (w/v). All experiments were conducted in triplicates. After the indicated time, the dry weight of BC pellicles was estimated as mentioned above.

### Response surface methodology approach

Box-Behnken design (Box and Behnken 1960) was employed to delimit the exact optimal level of each key determinant (independent variable) in respect to the decisive level of the response (dry weight of BC pellicle). Furthermore, all prospective interactions among the independent variables and their significant inferences on the response were well-modeled by a polynomial equation from the second order (Eq. 1). Three independent variables: glycerol concentration, inoculum size, and incubation time, derived from OVAT output, were tested. A matrix with fifteen trials was generated by Minitab 17.0 software. Each independent variable was assigned in each trial in one of three codes: − 1 (low level), 0 (center level), and + 1 (high level) as portrayed in Table 1.

1 $$ Y = \beta_{0} + \mathop \sum \limits_{i = 1}^{k} \beta_{i} x_{i} + \mathop \sum \limits_{i = 1}^{k} \beta_{ii} x_{i} x_{i} + \mathop \sum \limits_{i = 1}^{k - 1} \mathop \sum \limits_{j = 2}^{k} \beta_{ij} x_{i} x_{j} + \varepsilon $$

**Table 1:** Box-Behnken matrix with three independent variables along with experimental values in terms of dry weight BC pellicle produced by *K. saccharivorans* strain HIJ12

Trial #	X1 (Glycerol)	X2 (Inoculum size)	X3 (Incubation time)	(Dry weight BC pellicle: g/160 mL)		Standardized residuals
				Exp. values	Predicted values	
1	0(1.5)	0(6)	0(7)	4.8	4.6	0.40
2	0(1.5)	− 1(2)	− 1(4)	2.5	2.3	0.37
3	− 1(0.5)	0(6)	− 1(4)	7.6	7.8	− 0.24
4	0(1.5)	1(10)	− 1(4)	7.4	6.6	1.50
5	− 1(0.5)	0(6)	1(10)	7.0	6.2	1.70
6	1(2.5)	0(6)	1(10)	3.9	3.8	0.24
7	1(2.5)	1(10)	0(7)	4.5	4.5	0.13
8	− 1(0.5)	1(10)	0(7)	6.6	7.3	− 1.30
9	1(2.5)	− 1(2)	0(7)	0.6	0.0	1.30
10	0(1.5)	0(6)	0(7)	4.5	4.6	− 0.05
11	− 1(0.5)	− 1(2)	0(7)	6.3	6.4	− 0.13
12	1(2.5)	0(6)	− 1(4)	0.0	0.9	− 1.70
13	0(1.5)	0(6)	0(7)	4.4	4.6	− 0.40
14	0(1.5)	1(10)	1(10)	5.5	5.7	− 0.40
15	0(1.5)	− 1(2)	1(10)	3.7	4.5	− 1.50

Values between in brackets are real values. X_1_: (v/v) mL/mL, X_2_: (v/v) mL/mL, X_3_: days

Exp values, Experimental values; Pred. values, Predicted values; BC, Bacterial cellulose

Growth conditions: 160 mL core BC production medium in 1L Erlenmeyer flask

where Y is the level of dry weight of dry BC pellicle (response), *x*_1_, *x*_2_, *x*_3_,… *x*_i_ are the independent variables imposing an impact on the response, *β*_0_ is the model intercept, *β*_i_ (i = 1, 2,…,k) is the linear estimate of the variable, *β*_ii_ (i = 1, 2,…,k) is the quadratic estimate of the variable, *β*_ij_ (i = 1, 2,…,k; j = 1, 2,…,k) is the cross-interaction estimate of the variable and € is the random error. For statistical calculations, each independent variable *x* was coded as *x*_i_ according to Eq. 2. where *X*_i_ is a dimensional coded value of the independent variable, *xi* is the real value of this variable at this coded value, *x*_o_ is the real value of this variable at the center point (zero level) and *Δx*_i_ is the step change value. Subsequently, the three coded values for each independent variable had three corresponding real values as shown in Table 1.

2 $$X_{i} = \frac{\left(x_{i} - x_{0}\right)}{\Delta x_{i}}$$

The settings of the other cultivation conditions were 28 °C, a pH 5.5, *G. soli* BWH (160 mL in 1L Erlenmeyer flask), alcohol (0.5% (v/v)), yeast extract (0.5% (w/v)) and 2 days inoculum age. All experimental trials were conducted in triplicates. The nature of stationary point, whether maximum or minimum or saddle, was analyzed by the sign of eigen values. When a saddle point is encountered, ridge analysis serves as the established statistical alternative (Myers, 1976), Using RSM package which is reachable at the Compre­hensive R Archive Network http://CRAN.R-project.org/package=rsm Lenth 2009. After the indicated time, the dry weight of BC pellicles was estimated as mentioned above.

### Purification of BC

#### Alkaline method

The pellicles of BC were harvested from the culture medium using sterile forceps and rinsed several times with distilled water to remove loosely attached cells and residual medium. The pellicles were then purified by alkaline treatment in 0.5–1.0 M NaOH at 80 °C for 1–2 h with gentle stirring to lyse bacterial cells and solubilize residual proteins, lipids, and medium components, as described previously (Anguluri et al. 2022). Following treatment, the pellicles were washed thoroughly with warm distilled water until neutral pH was achieved. When necessary, a bleaching step was performed using 5–10% H_2_O_2_ for 30–60 min under gentle agitation, followed by extensive washing. Finally, purified BC pellicles were dried at 40–60 °C until constant weight was obtained.

#### Gradient ethanol method

BC was purified using gradient ethanol-dependent method according to a previously reported procedure (Aditiawati et al. 2023). Washed pellicles were sequentially immersed in ethanol solutions of increasing concentrations (10–90%, v/v) for 15 min each on a shaker at room temperature. After that, all samples were immersed twice in 100% ethanol for 15 min under the same conditions. The purified pellicles were stored at − 80 °C until further analysis. Prior to characterization, the samples were freeze-dried to preserve structure and eliminate residual moisture. The efficiency of purification was evaluated using scanning electron microscopy (SEM).

### Characterization of BC

#### Fourier transform infra-red (FTIR)

FTIR spectroscopy was used to identify the chemical structure of the BC (Ashori et al. 2012; Altunordu et al. 2025). Infrared absorption spectra were obtained from 4000 to 400 cm ^−1^ using KBr pellets in a PerkinElmer spectrometer, model 2000. Baseline correction and normalization were applied consistently across all spectra.

#### X-ray diffraction (XRD)

The crystal structure of the BC, in terms of crystalline index and crystallite size, was analyzed by using an X-Ray diffractometer using a Bruker D2 Phaser diffractometer (Madison, WI, USA) at room temperature, with CuKα radiation generated at 1.5406 Å wavelength, operated at 40 kV and 8 mA (Vasconcelos et al. 2017; Thorat and Dastager 2018). The crystallinity was calculated according to Segal formula (Segal et al. 1959), shown in Eq. 3.

3 $$ CrI = \frac{{\left( {CI_{200} - I_{am} } \right)}}{{I_{200} }} \times 100 $$

I_200_: maximum diffraction intensity of the crystallization peak around 2θ = 22°.I_am_: minimum diffraction intensity of the crystallization peak around 2θ = 18°.

#### Scanning electron microscope analysis (SEM)

The micromorphology of BC was investigated using a SEM (JEOL JSM-IT200, Japan) at an accelerating voltage of 5–15 kV according to a previously reported procedure (Altunordu et al. 2025; Fatima et al. 2023).

#### Thermogravimetric analysis (TGA) and differential scanning calorimetry (DSC)

Thermogravimetric analysis of dry BC was performed with a thermal weight analyzer (TG/DTA7200, Hitachi Technology Co. Ltd, Japan). The temperature range, ranging between 30 and 800 ℃. Thermogravimetric (TGA) and differential scanning calorimetry (DSC) curves were recorded simultaneously to monitor weight loss and thermal transitions.

#### Tensile properties

The mechanical strength of BC was determined according to a previously reported procedure (Altunordu et al. 2025; Wang et al. 2021; Hsieh et al. 2008). The tensile characteristics of BC was done by using a texture meter (TA.XT Plus, Stable Micro Systems Technology Co., Ltd, UK) with a mechanical probe of 150 N. The tensile properties of BC were investigated by TA-TGA stretching fixture, and the thickness of BC was measured at 1.5 ± 0.1 mm, the width and the length were measured at 10 and 20 mm, respectively. Ultimate tensile strength (UTS, MPa) was calculated by dividing the ultimate force by the specimen’s cross-sectional area.

### Hydrophilic properties of BC

#### Determination of wet and dry weights of BC

After production, BC pellicles were isolated and washed thoroughly with distilled water. Excess surface water was removed by placing the pellicles between two filter papers prior to weighing. The wet weight (m_wet_) was recorded immediately. Samples were then dried at room temperature (RT) for 24–96 h, and the corresponding dry weights (m_RT−dry_) were recorded at different time intervals. For complete water removal, pellicles were frozen at − 80 °C for 24 h and subsequently freeze-dried for 72 h to obtain the final freeze-dried weight (m_FD−dry_).

#### Moisture content (MC) and water holding capacity (WHC)

The moisture content and water holding capacity of BC were determined according to two previously reported procedures (Fang and Catchmark 2014; Shezad et al. 2010), respectively (Eqs. 4 and 5).

4 $$ MC \left( \% \right) = \frac{{m_{wet} - m_{FD - dry} }}{{m_{wet} }} \times 100 $$

where, m_wet_ is the weight after washing and surface water removal, and m_FD-dry_ is the freeze-dried weight.

5 $$ WHC \left( \% \right) = \frac{{m_{wet} - m_{RT - dry} }}{{m_{FD - dry} }} \times 100 $$

where, m_RT-dry_ is the RT-dried weight at 48–96 h and m_FD-dry_ is the freeze-dried weight.

#### Water retention rate (WRR) and Rehydration ratio (RR)

The water retention rate and rehydration rate were determined according to a previously reported procedures (Shezad et al. 2010; Chang and Chen 2016) (Eqs. 6 and 7).

6 $$ WRR \left( g \right) = m_{RT - dry} - m_{FD - dry} $$

where, m_RT-dry_ is the RT-dried weight at 48–96 h and m_FD-dry_ is the freeze-dried weight.

7 $$ RR \left( \% \right) = \frac{{m_{rwet} - m_{FD - dry} }}{{m_{wet} - m_{FD - dry} }} \times 100 $$

where, m_FD-dry_ represents the freeze-dried weight, and m_rwet_ is the weight measured after immersing the sample in deionized water for 48–96 h.

#### Porosity

The porosity of BC was determined according to a previously reported method (Hossen et al. 2020) (Eq. 8).

8 $$ Porosity \left( \% \right) = \frac{wet weight - dry weight}{{wet weight - weight in water}} \times 100 $$

#### Statistical analysis

GraphPad Prism 8.0 was used to analyze the OVAT data and estimate the significance at *P*-value < 0.05 and draw all figures related to OVAT data. Minitab 17.0 was used to generate Box-Behnken design. The RSM package is reachable at the Compre­hensive R Archive Network (http://CRAN.R-project.org/package=rsm) Lenth 2009, was employed in this study for performing, multiple non-linear regression, canonical, and ridge analyses. Statistica software 13.0 was used to portray the three-dimensional surface plots describing the relationship among the independent variables and the response.

## Results

### *Komagataeibacter* sp. strain HIJ12 EMCCN-4085: identification and Phylogeny

**Fig. 1:**
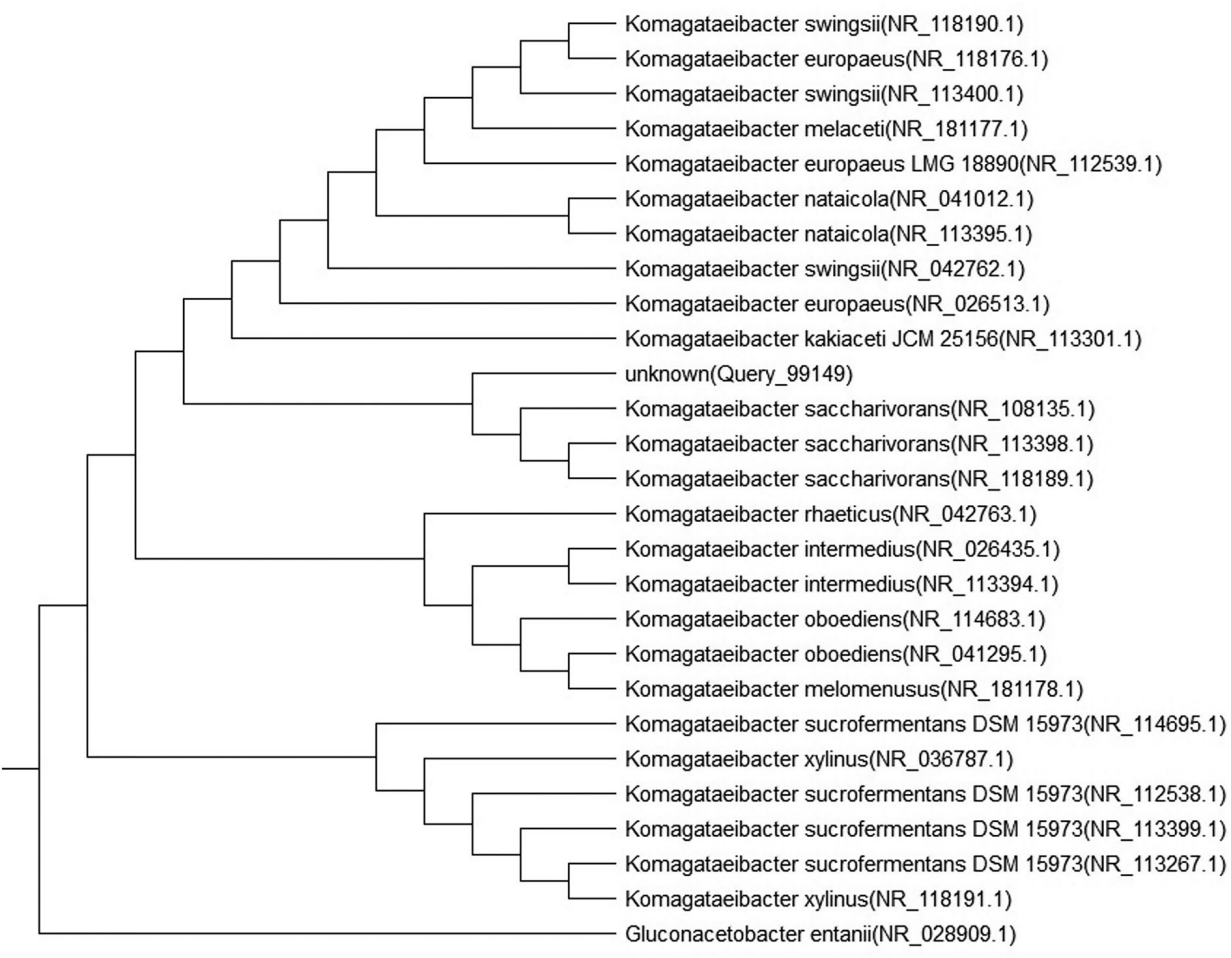
Maximum Likelihood phylogenetic tree (MEGA 11.0) elucidating the phylogenetic relationship between 16S rRNA sequence of the BC-producing bacterial isolate namely *Komagataeibacter* sp. strain HIJ 12 EMCCN4085 and other 16S rRNA nucleotide sequences of closely related *Komagataeibacter* spp. Bootstrap values are indicated by numbers on branch nodes (1000 re-samplings)

BLASTN search sequence analysis showed that the 16S rRNA nucleotide sequence of the BC-producing bacterial isolate did match with several hits of *Komagataeibacter* spp. (e.g., NR_108135.1.1: *K. saccharoivorans* strain LMG 1582 with 99.9%, NR_112228.1: *K. saccharoivorans* strain LMG 1582 with 99.9%, 113398.1, *K. saccharoivorans* strain JCM 25121 with 99.9%, NR_179977.1: *K*. *diospyri* strain MSKU9 with 98%, and NR_112539.1: *K. europaeus* with 99%). After performing multiple sequence alignment between 16S rRNA sequence of the BC-producing bacterial isolate (query) and other hits of 16S rRNA nucleotide sequence of closely related *Komagataeibacter* spp., showing high sequence identity with the query sequence based on BLASTN output, the affiliation of the investigated bacterial isolate to *Komagataeibacter* sp. was determined using a Maximum Likelihood phylogenetic tree elucidated in Fig. 1. The strain was designated HIJ12 in compliance with GenBank submission guidelines. In addition, the bacterial strain was deposited in EMCCN at the National Research Center in Cairo, Egypt under the accession number EMCCN-4085. Hence, the bacterial strain was finally assigned to *Komagataeibacter* sp. Strain HIJ12 EMCCN-4085.

### Statistically optimized BC synthesis by *Komagataeibacter* sp. strain HIJ12 EMCCN-4085

#### Key determinants controlling the production of BC

To identify the physicochemical variables most influential on BC biosynthesis, nine parameters were examined sequentially using an OVAT approach, with each optimized condition fixed before proceeding to the next variable. This sequential strategy established a robust baseline medium and cultivation setup prior to multivariate statistical optimization.

**Fig. 2:**
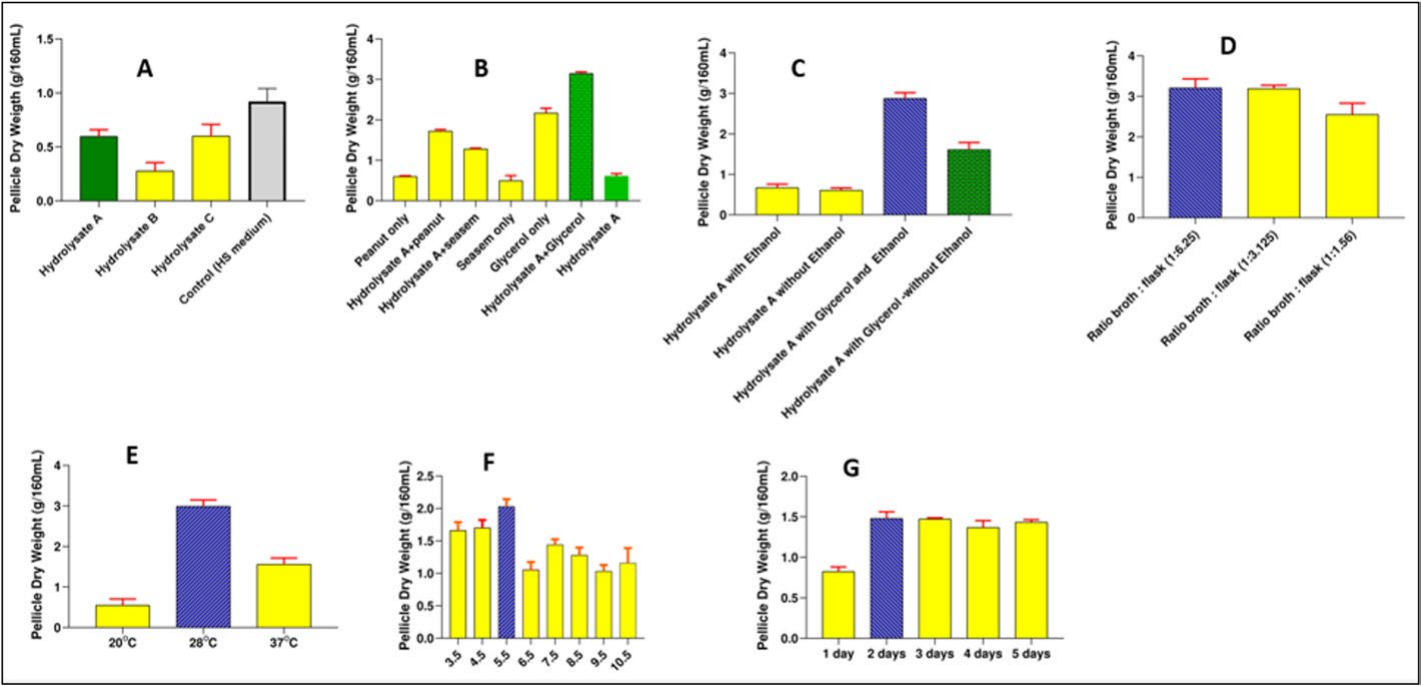
Screening the effect of seven independent variables on the synthesis of BC by *Komagataeibacter* sp. strain HIJ 12 EMCCN4085 via OVAT approach. **A** Effect of using different BWHs, **B** effect of using different carbon sources, **C** effect of ethanol, **D** effect of using aeration ratios (volume of broth: volume of Erlenmeyer flask), **E** effect of using different incubation temperature, **F** effect of using different pH(s) of the production medium, and **G** effect of using different inoculum age. Values are the mean of three readings ± standard error. Growth conditions were 160 mL core BC production medium in 1L Erlenmeyer flask

Among the three BWHs evaluated, that prepared using *G. soli* AM6 EMCCN3074 supported significantly higher BC pellicle dry weight (0.6 ± 0.1 g L^−1^; *P* < 0.05) than hydrolysates derived from *P. chrysogenum* or *A. oryzae*, with no significant difference detected between the latter two (Fig. 2A). Accordingly, *G. soli* BWH was selected as the production medium for all subsequent experiments.

Screening of carbon sources revealed that glycerol supplementation alone produced a significantly higher BC pellicle dry weight (2.2 ± 0.12 g/160 mL) than either peanut or sesame oil (Fig. 2B). Further, combining glycerol with *G. soli* BWH yielded a synergistic increase in BC production (3.14 ± 0.04 g/160 mL), surpassing yields obtained with peanut or sesame supplementation under the same hydrolysate background. This glycerol–hydrolysate combination was therefore selected as the carbon source platform for subsequent experimentation.

The addition of ethanol to the glycerol–hydrolysate blend significantly enhanced BC pellicle dry weight to 2.9 ± 0.12 g/160 mL (*P* < 0.05), exceeding all other supplementation combinations tested (Fig. 2C), consistent with the established role of ethanol as a metabolic modulator supporting cellulose biosynthesis in *Komagataeibacter*.

Regarding aeration, a broth-to-flask volume ratio of 1:6.3 yielded significantly higher BC production (3.2 ± 0.22 g/160 mL) than the 1:1.56 ratio (Fig. 2D). No significant difference was detected between ratios of 1:6.3 and 1:3.13; consequently, the 1:6.3 ratio was adopted to ensure adequate oxygen transfer in subsequent experiments.

Incubation at 28 °C produced significantly higher BC pellicle dry weight (3.0 ± 0.16 g/160 mL) than either 20 °C or 37 °C (Fig. 2E), establishing 28 °C as the optimal temperature for *Komagataeibacter* sp. strain HIJ12 EMCCN-4085 under the present culture conditions.

Supplementation with either organic or inorganic nitrogen sources did not significantly affect BC pellicle dry weight (Fig. S1), indicating that the *G. soli* BWH inherently provided sufficient nitrogen and growth-supporting nutrients to sustain cellulose biosynthesis without additional nitrogen supplementation.

Among all parameters screened, initial medium pH exerted the strongest and most decisive influence on BC yield, with a sharp optimum observed at pH 5.5 producing significantly higher BC pellicle dry weight than any other pH tested (Fig. 2F). Yield declined steeply upon deviation above or below this value, indicating a narrow effective pH window characteristic of *Komagataeibacter* static cultures.

A two-day inoculum age produced the highest BC pellicle dry weight (1.5 ± 0.1 g/160 mL; *P* < 0.05), significantly exceeding that obtained with a one-day inoculum, with no statistically significant improvement observed at longer inoculum ages (Fig. 2G). These results indicate that a two-day inoculum provides the optimal physiological state for pellicle establishment at the air–liquid interface.

Collectively, OVAT screening defined the following optimized baseline for BC production: *G. soli* BWH (160 mL/1 L Erlenmeyer flask) supplemented with glycerol (4 mL/160 mL) and ethanol (0.8 mL/160 mL), incubated at 28 °C, pH 5.5, using a 2-day-old inoculum at an aeration ratio of 1:6.3. Among the screened variables, pH was fixed at 5.5 rather than carried forward to RSM, as its sharp optimum and narrow effective range preclude meaningful polynomial modeling. Similarly, inoculum age was fixed at two days rather than included in the RSM design, as BC yield plateaued beyond this point with no statistically significant improvement at longer inoculum ages, indicating a threshold-type response rather than a continuous quantitative relationship across the tested range, a response pattern incompatible with the quadratic polynomial modeling underlying BBD. In contrast, glycerol concentration, inoculum size, and incubation time, each exhibiting continuous, quantitative responses across a practical operating range, were selected as independent variables for Box–Behnken design-based Response Surface Methodology to precisely define their optimal levels and interaction effects on BC yield.

#### Attaining optimized conditions

Box–Behnken design-based Response Surface Methodology was applied to precisely define the optimal levels of three key independent variables, glycerol concentration (X_1_), inoculum size (X_2_), and incubation time (X_3_), with respect to BC pellicle dry weight as the response. The experimental design matrix, including real and coded values of the independent variables alongside the corresponding experimental and predicted response values, is presented in Table 1. The regression coefficients and statistical parameters derived from multiple non-linear regression analysis are summarized in Table 2.

**Table 2:** Regression summary for full polynomial equation of Box-Behnken design for BC pellicle production by Komagataeibacter saccharivorans strain HIJ12

Model term	β co-efficient	*t*-value	*P*-value	Confidence level (%)
Intercept	4.6	8.94	0.0003	99.97****
X1	− 2.3	− 7.45	0.0007	99.93***
X2	1.4	4.35	0.007	99.26**
X3	0.32	1.03	0.349	–
X1X2	− 0.08	− 0.18	0.866	–
X1X3	0.06	0.12	0.908	–
X2X3	0.17	0.38	0.723	–
X1X1	0.90	2.04	0.097	90.26*
X2X2	1.13	2.6	0.050	94.975**
X3X3	− 0.79	− 1.8	0.129	–

^****^ significant at *P* = 0.0001, *** significant at *P* = 0.001,** significant at *P*-value = < 0.05, and * significant at *P*-value = 0.1

ANOVA revealed a model *F*-value of 9.96 and a *P*-value of 0.01, confirming the statistical significance of the model and indicating only a 1% probability that this *F*-value arose from random noise. Model adequacy was evaluated using three statistical estimators: the multiple correlation coefficient (*R*), the coefficient of determination (*R*^2^), and the adjusted *R*^2^. The model yielded a multiple *R* value of 0.97, reflecting a strong correlation between experimental and model-predicted response values. The *R*^2^ value of 0.95 indicates that 95% of the total variation in BC yield was accounted for by the model, encompassing all main effects, cross-interactions, and quadratic terms. The adjusted *R*^2^ of 0.85 further confirms that 85% of the response variability is explained by the model after correction for the number of predictors, collectively demonstrating a satisfactory model fit.

The relationship between the independent variables and the response was described by the following second-order polynomial equation (Eq. 9) expressed in coded values:

9 $$ \begin{aligned} Y = & 4.6 - 2.3X_{1} + 1.4X_{2} + 0.32X_{3} \\ & - 0.08X_{1} X_{2} + 0.06X_{1} X_{3} + 0.17X_{2} X_{3} \\ & + 0.9X_{1} X_{1} + 1.13X_{2} X_{2} - 0.79X_{3} X_{3} \\ \end{aligned} $$

Multiple non-linear regression analysis identified glycerol concentration (X_1_) and inoculum size (X_2_) as the only variables exerting statistically significant effects on BC pellicle dry weight production in both their linear and cross-interaction terms (*P* < 0.05), as detailed in Table 2. Solving Eq. 9 by differentiation yielded a predicted stationary point at coded values of X_1_ = − 0.27, X_2_ = 0.66, and X_3_ = 1.5, corresponding to a predicted BC pellicle dry weight of 5.6 g/160 mL. Canonical analysis was subsequently performed using the RSM package to determine whether this stationary point represents a maximum, minimum, or saddle point (Table 3A).

**Table 3:** Steepest ascent path for estimated ridge using ridge analysis for the response of BC pellicle production by K. saccharivorans strain HIJ12

Distance	X1	X2	X3	Predicted BC Pellicle dry weight (g/160 mL)
0	0.0	0.0	0.0	4.6
0.5	− 0.44	0.22	− 0.08	5.8
1	− 0.85	0.35	− 0.39	7.1
1.5	− 1.21	0.43	− 0.77	8.6
2.0	− 1.54	0.51	− 1.17	10.3

**Fig. 3:**
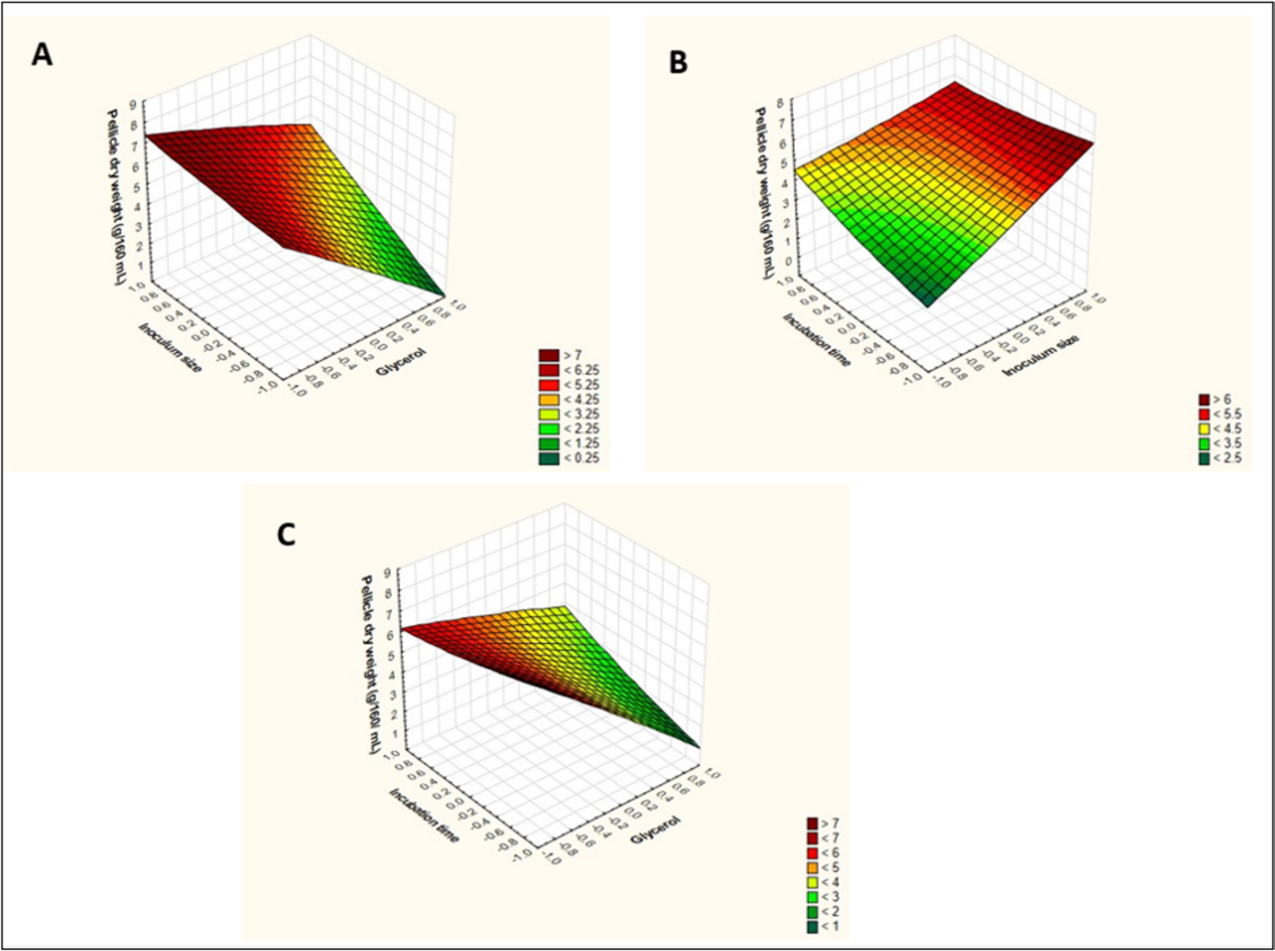
Three- dimensional surface plots generated by Statistica 12.0 deciphering the relationship between each two independent variables in relation to the response at fixed value for the third independent variable. **A** X1 and X2 in relation to the response (BC dry weight pellicle, **B** X2 and X3 in relation to the response (BC dry weight pellicle, and **C** X1and X3 in relation to the response (BC dry weight pellicle. Growth conditions were 160 mL core BC production medium in 1L Erlenmeyer

The canonical analysis yielded eigenvalues of λ_1_ = 0.63, λ_2_ = 0.43, and λ_3_ = − 0.91 for X_1_, X_2_, and X_3_, respectively. The magnitude and sign of each eigenvalue describe the curvature of the response surface in the corresponding direction, with positive and negative values indicating upward and downward curvature, respectively (Myers 1976). The larger the absolute eigenvalue, the more pronounced the curvature of the response in that direction (Myers 1976). The mixed signs of the obtained eigenvalues confirmed that the stationary point is a saddle point rather than a true maximum or minimum (Fig. 3A–C), with the most pronounced curvature occurring in the direction of X_1_ (λ_1_ = 0.63), followed by X_2_ (λ_2_ = 0.43), consistent with the regression results identifying these two variables as the primary drivers of BC yield.

Since the coded value of X_3_ at the stationary point (1.5) falls outside the design constraints, traversing the response surface along the canonical path in either direction was not statistically justified. Ridge analysis was therefore applied as the appropriate alternative strategy, moving along the path of steepest ascent from the design origin to locate the maximum feasible response within the design space (Table 3B). Progression along the rising ridge produced a continuous elevation in the predicted response without reaching a plateau; however, predictor combination sets at ridge distances exceeding 1.0 fell outside the design constraints. The predictor combination set at a ridge distance of 1.0 was therefore selected for experimental validation.

At a ridge distance of 1.0, the optimal coded values were X_1_ = − 0.84, X_2_ = 0.35, and X_3_ = − 0.39, corresponding to real values of 0.67 mL glycerol/160 mL, 7.4 mL inoculum size/160 mL, and 5.8 days incubation time, respectively, with a model-predicted BC pellicle dry weight of 7.1 g/160 mL. Experimental validation under these conditions yielded a BC pellicle dry weight of 6.2 g/160 mL, representing a model validation accuracy of 88.3%. The optimized yield corresponds to a tenfold enhancement relative to the pre-optimization baseline yield of 0.6 g/160 mL, equivalent to a final BC dry weight productivity of 39 g dry weight L⁻^1^ by *Komagataeibacter* sp. strain HIJ12 EMCCN-4085.

### Characteristics of hydrolysate-based BC

**Fig. 4:**
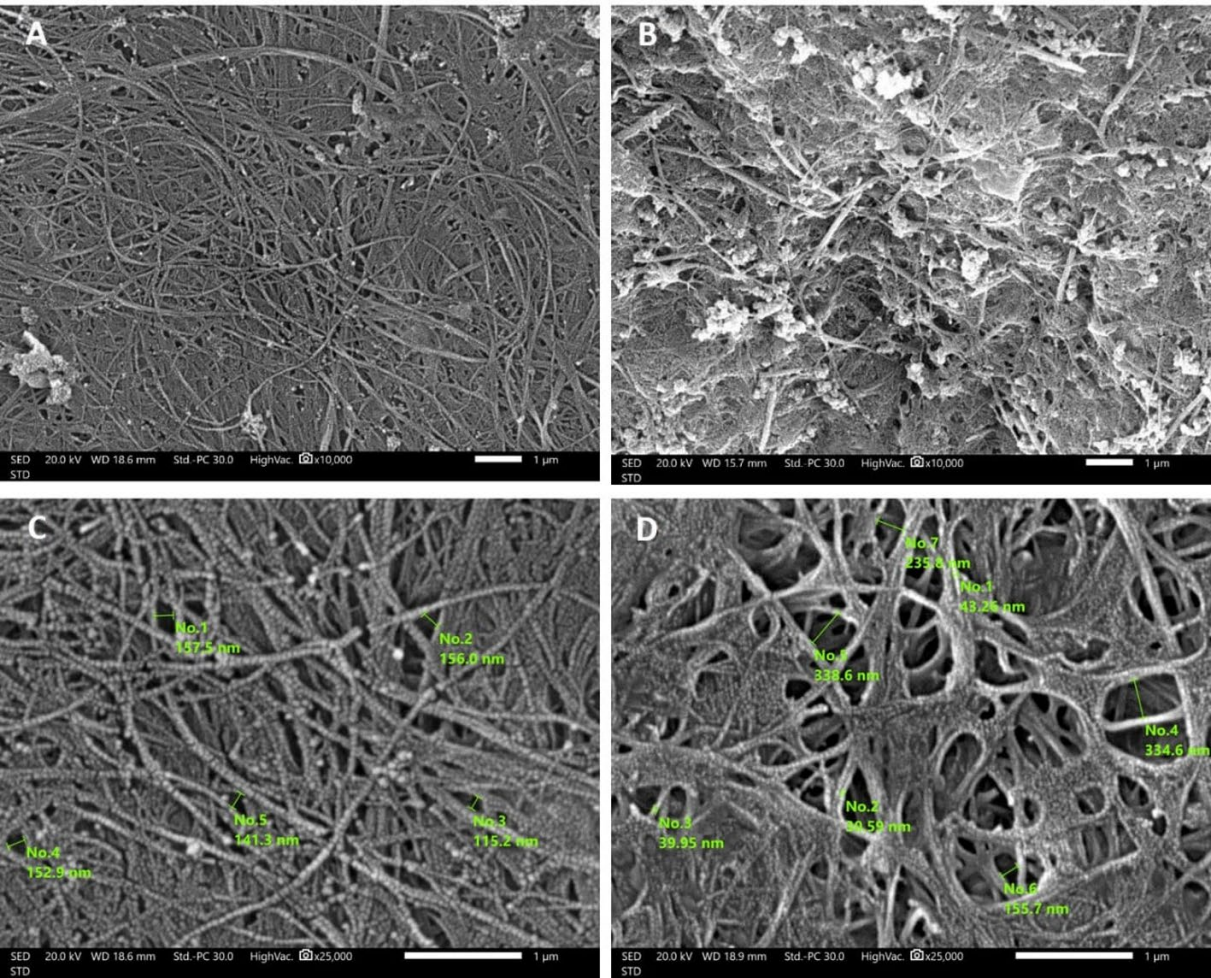
Scanning Electron Microscopy (SEM) images of BC purified using different methods at 10,000× and 25,000× . (**A**, **B**) HS-based BC and hydrolysate-based BC, respectively, after alkaline purification. (**C**, **D**) HS-based BC and hydrolysate-based BC, respectively, following incorporation of a gradient ethanol purification step after alkaline treatment showing variations in pore sizes

The conventional alkaline purification method retained BC (HS-based or hydrolysate-based BC) with non-distinct nanofibrillar structure (Fig. 4A, B). In stark contrast, the ethanol gradient purification method yielded a much clearer fibrillar structure than that obtained via the alkaline method (Fig. 4C, D). The pore size of hydrolysate -based BC was larger than that of HS-based BC (Fig. 4C, D).

**Fig. 5:**
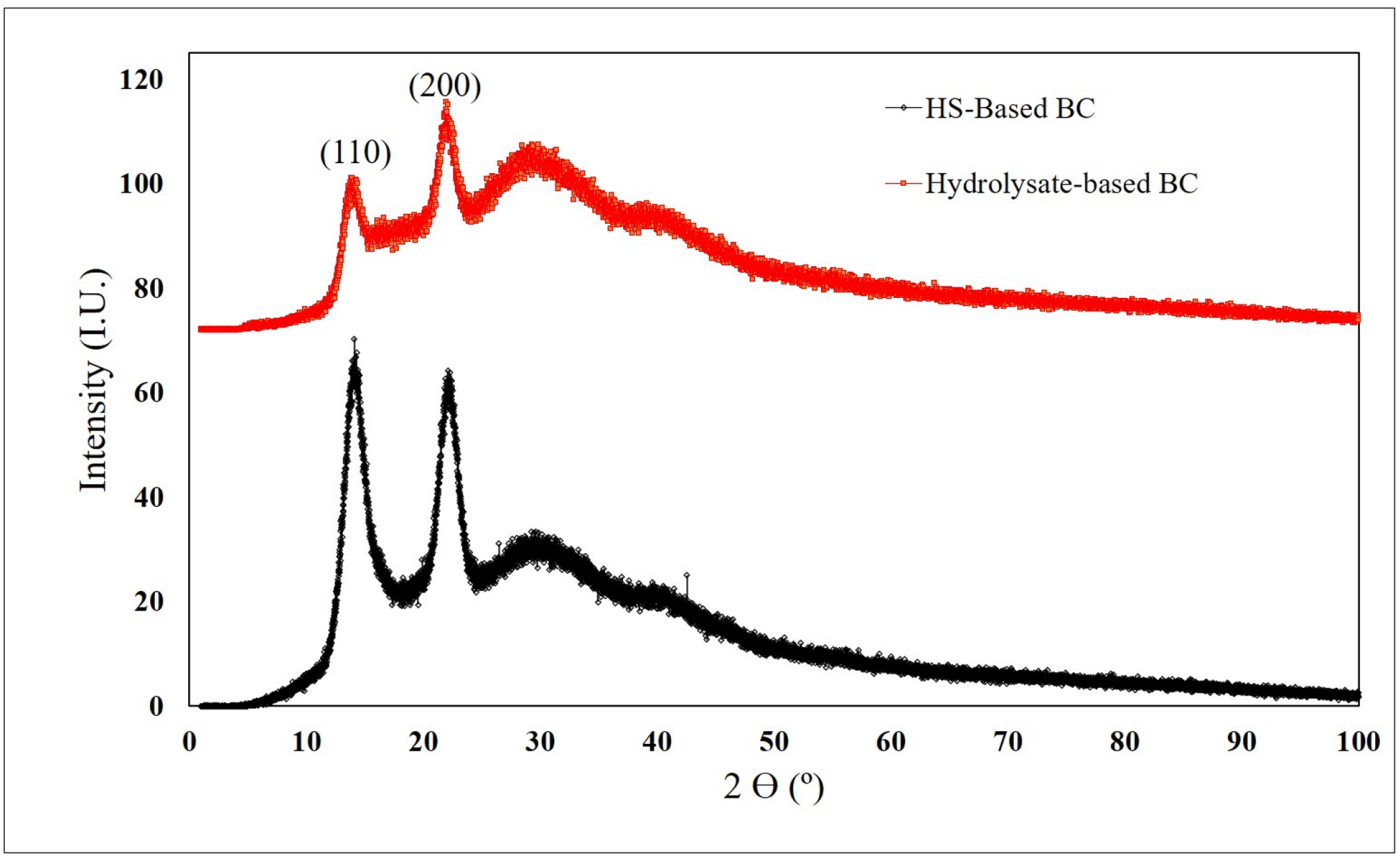
X-ray diffraction (XRD) patterns of BC produced by standard HS medium (black line) and BWH-based medium (red lines). Diffractograms were baseline-corrected and normalized for direct comparison

The cellulose I crystalline structure for both BC types was confirmed via XRD analyses, showing uniform diffraction peaks of ~ 14.7–14.8° (110) and ~ 22.4–22.5° (200) (2θ) (Table 4 and Fig. 5). However, the intensity (I_200_) of BC varied from 62.5 a.u., for HS-based BC, to 43.5 a.u for hydrolysate -based BC. In stark contrast, the amorphous intensity (I_am_) value of 19.3 a.u. was identical for both BC types. Based on Segal’s method, the CrI was 69 and 54% for HS-based BC and hydrolysate-based BC, respectively. Present findings evidence that HS-based BC and hydrolysate-based BC favour ordered cellulose fibrils with high crystallinity and heterogenous fibrils structures, respectively.

**Table 4:** X-ray diffraction (XRD) parameters of HS-based BC and hydrolysate-based BC

Sample	Main peaks (2θ, °)	Miller indices	I_200_ (a.u.)	I_am_ (a.u.)	CrI (%)
SH-based BC	14.8, 22.5	(110), (200)	62.5	19.27	69
Hydrolysate-based BC	14.7, 22.4	(110), (200)	43.5	19.9	54

TGA curves analyses showed three stages for thermal stabilities: initial decomposition, major decomposition, and final weight with three distinctive temperature ranges for both BC types (Fig. S2). The initial decomposition occurred at ~ 165 °C, with mass loss of ~ 4.9% and at ~ 188 °C, with mass loss of ~ 9.8% for HS-based BC and hydrolysate -based BC, respectively. The major decomposition resulted in ~ 84.5% mass loss at ~ 165–330 °C and ~ 73.0% mass loss at ~ 188–325 °C, for HS-based BC and hydrolysate -based BC, respectively. In the final stage of composition, the remaining mass was ~ 9.6 and ~ 16% at 800 °C for HS-based BC and hydrolysate -based BC, respectively. The DSC profile for both BC types was displayed in Fig. S2. Both BC types exhibited almost quite similar DSC profile with an endothermic event near 100 °C (moisture evaporation) and an exothermic peak around 330 °C. However, the decomposition peak was slightly shifted in hydrolysate -based BC compared to HS-based BC. This would in turn reflect and verify the difference in cellulose nature and organization in both BC types.

The FTIR spectral pattern for both BC types was illustrated in Fig. S3. Collectively, the FTIR spectral profile revealed almost quite similar pattern for both types of BC. A broad absorption band at ~ 3300–3400 cm^−1^ was detected in both samples, corresponding to O–H stretching vibrations of hydrogen-bonded hydroxyl groups. C–H stretching bands were observed near 2900 cm^−1^. In the carbonyl region (~ 1730–1650 cm^−1^), weak absorption signals were identified, likely associated with trace carboxyl groups or bound water. The region between 1200 and 1000 cm^−1^ showed prominent bands attributable to C–O–C stretching of glycosidic linkages and C–O vibrations of the pyranose ring. Skeletal vibrations and out-of-plane deformations were also evident below 700 cm^−1^. In essence, these features confirmed the cellulose I structure and indicated that both media yielded chemically consistent BC.

The tensile performance of both BC types was presented in Table 5. HS-based BC films (cross-sectional area: 200 mm^2^) exhibited an ultimate force of 16.32 N, corresponding to an ultimate tensile strength of 0.08 MPa, with a break distance of 20.1 mm. In contrast, hydrolysate-based BC membranes (cross-sectional area: 100 mm^2^) showed markedly reduced mechanical strength, with an ultimate force of 4N, tensile strength of 0.04 MPa, and break distance of 13.6 mm. These results demonstrate that HS-based BC possesses approximately twice the tensile strength and ~ 48% higher elongation before failure compared to hydrolysate-based BC, indicating a mechanically more robust fibrillar network in the control sample.

**Table 5:** Mechanical properties of BC membranes

Sample	Cross-sectional area (mm^2^)	Ultimate force (N)	Ultimate tensile strength (MPa)	Break distance (mm)
HS-based BC	200	16.32	0.08	20.1
Hydrolysate-based BC	100	4.00	0.04	13.6

The hydrophilic properties of both types of BC were summarized in Table 6. Both BC types exhibited high moisture content, with values of 96.7 ± 1.2% for hydrolysate-based BC and 97 ± 0.6% for HS-based BC. The water holding capacity was comparable between the two samples, measuring 49 ± 4% and 49.7 ± 16.5% for hydrolysate- and HS-based BC, respectively. A marked difference was observed in water retention rate, with hydrolysate-based BC (0.013 ± 0.01 g) displaying significantly higher retention compared to HS-based BC (0.003 ± 0.01 g). The rehydration ratio remained similar across the two groups, with values of 12 ± 1.7% for hydrolysate-based BC and 12.7 ± 2.1% for HS-based BC. Notably, porosity differed between the samples, where hydrolysate-based BC exhibited a higher porosity of 41 ± 1.2%, compared to 28 ± 6.1% for HS-based BC.

**Table 6:** Hydrophilic properties of HS-based BC and hydrolysate-based BC

Parameter	Hydrolysate-based BC	HS-based BC
Moisture Content (%)	96.7 ± 1.2	97 ± 0.6
Water Holding Capacity (%)	49.0 ± 4.00	49.7 ± 16.5
Water Retention Rate (g)	0.01 ± 0.02	0.003 ± 0.02
Rehydration Ratio (%)	12.0 ± 1.7	12.7 ± 2.1
Porosity (%)	41 ± 1.2	28.0 ± 6.1

Values are the mean of three readings ± SD

## Discussion

Bacterial cellulose (BC) formation by *Komagataeibacter* is governed by coupled nutritional and physical constraints. Under static culture, cellulose is synthesized predominantly at the air–liquid interface, so BC productivity depends not only on carbon availability and precursor supply but also on oxygen transfer and the ability of cells to establish a stable surface biofilm. Accordingly, we used a two-stage strategy: (i) one-variable-at-a-time (OVAT) screening to define a robust baseline medium and cultivation setup, and (ii) response-surface optimization to quantitatively refine the most influential variables within a feasible operating window. The discussion below places the observed screening trends in the context of prior waste-based BC systems and known physiological features of *Komagataeibacter*.

Among the three BWHs evaluated, that prepared using *G. soli* AM6 EMCCN3074 supported the highest initial BC yield (0.6 g L^−1^) prior to any targeted supplementation, reflecting the importance of hydrolysate composition, particularly the balance between readily assimilable sugars, oligosaccharides, and growth-supporting nutrients, in determining early BC productivity. Similar feedstock-dependence has been documented in other waste-based BC systems; brewing by-product extract, for instance, enabled approximately 4.0 g L^−1^ BC using *K. rhaeticus* UNIWA AAK2 at pH 6 and 30 g L^−1^ sugars after medium adjustment (Tsouko et al. 2023). The comparatively lower baseline yield observed here is consistent with a non-optimized screening-stage medium, underscoring the necessity of targeted carbon source reinforcement to redirect metabolic flux toward cellulose biosynthesis.

The synergistic increase in BC yield observed upon combining glycerol with the *G. soli* hydrolysate (3 g/160 mL) relative to glycerol alone (2.2 g /160 mL) suggests that the hydrolysate matrix contributes growth-supporting cofactors and oligomeric carbon species that complement the readily metabolizable glycerol co-substrate. The stimulatory effect of glycerol on *Komagataeibacter* BC production is well established; *K. rhaeticus* PG2, for example, produced approximately 8.7 g L^−1^ on HS medium supplemented with 3% (w/v) glycerol under static culture (Thorat and Dastager 2018). A plausible physiological basis is that glycerol sustains carbon flux toward cellulose precursors while attenuating periplasmic glucose oxidation to gluconic acid, a reaction that drives medium acidification and impairs pellicle formation under static conditions (Montenegro-Silva et al. 2024). The glycerol response observed in the present study is therefore consistent with both prior performance reports and the known sensitivity of BC biosynthesis to carbon partitioning and acid stress.

Ethanol supplementation further enhanced BC yield to 2.9 g /160 mL, consistent with its established role as a metabolic modulator in acetic-acid bacteria. In *Acetobacter xylinum* (currently *Komagataeibacter xylinus*), ethanol at approximately 1–3% (v/v) enhanced BC production through metabolic shifts favoring cellulose biosynthesis pathways (Yunoki et al. 2004), and proteomics-based evidence indicates that ethanol influences cellular redox balance and carbon partitioning in ways that sustain cellulose formation under static culture conditions (Fei et al. 2023). The modest but significant stimulation observed here upon low-dose ethanol addition to the glycerol–hydrolysate blend is mechanistically consistent with these reports.

The plateau-type response observed for aeration, wherein increasing the broth-to-flask ratio from 1:1.6 to 1:6.3 raised BC yield to 3.2 g /160 mL with no significant further gain beyond 1:3.125, indicates that oxygen limitation at the air–liquid interface was effectively alleviated within this range. This finding aligns with oxygen-focused BC studies demonstrating that supplying air at 6.3 L min^−1^ to the medium surface increased BC yield by approximately 25% while preserving film quality (Park et al. 2003), and with classical static-culture work showing that controlling gaseous oxygen tension between 10–15% O_2_ significantly influenced cellulose formation and membrane properties (Watanabe and Yamanaka 1995). Collectively, these results reinforce the primacy of interfacial oxygen availability as a driver of pellicle establishment in strictly aerobic *Komagataeibacter* cultures.

The optimal incubation temperature of 28 °C is consistent with the broadly reported thermal window for *Komagataeibacter* static BC production. *K. rhaeticus* K23 maximized BC at 32 °C under Taguchi-optimized conditions (9.1 ± 0.7 g L^−1^) (Uğurel and Öğüt 2024), and *K. xylinus* peaked at 30 °C with 8.5 g L^−1^ in a waste-based system (Yilmaz and Göksungur 2024), indicating that the effective thermal range spans approximately 28–32 °C across strains and media. The slightly lower optimum observed here (28 °C) may reflect the thermosensitivity of specific enzymatic steps in cellulose biosynthesis under the heterogeneous hydrolysate medium composition.

The threshold-type response of BC yield to inoculum age, peaking at two days with no significant improvement thereafter, indicates that the physiological state of the inoculum critically governs early pellicle establishment at the air–liquid interface. A comparable pattern has been reported for *Gluconacetobacter hansenii*, where a three-day inoculum sustained high cellulose production across repeated batches, suggesting that a mid-exponential physiological state stabilizes productivity (Park et al. 2003). Similarly, kombucha-based BC studies demonstrated that low inoculum fractions (2.5–5%) significantly reduced yield, confirming that inoculum condition is a primary productivity determinant (Guimarães et al. 2024). The absence of yield improvement beyond two days in the present system most likely reflects the onset of nutrient depletion or metabolic shift toward stationary phase, rendering older inocula less effective at rapidly establishing the surface pellicle.

The absence of a significant nitrogen supplementation effect is attributable to the inherent nutritional richness of the *G. soli* BWH, which appears to provide sufficient assimilable nitrogen and growth factors to sustain *Komagataeibacter* cellulose biosynthesis without additional supplementation. A parallel conclusion was reached in HS-type media already containing yeast extract and peptone, where altering the nitrogen source produced little additional effect on BC yield because nitrogen was not the limiting factor (Santos et al. 2013). This contrasts with nutritionally unbalanced waste substrates, such as waste fig medium, where yeast extract supplementation remained significant, indicating that some carbon-rich wastes require nitrogen fortification to support high BC formation (Yilmaz and Göksungur 2024). The nitrogen sufficiency of the *G. soli* hydrolysate therefore represents an additional practical advantage of microbially generated BWHs over simple chemical or aqueous extracts.

Among all screened variables, initial medium pH exerted the most decisive influence on BC yield, with a sharp optimum at pH 5.5 and steep yield decline upon deviation in either direction. This narrow effective pH window is well documented for static *Komagataeibacter* cultures; *K. rhaeticus* K23 also maximized BC at pH 5.5 (approximately 9.1 g L^−1^) (Uğurel and Öğüt 2024), while a waste-fig-based system peaked slightly higher at pH 6.1 (approximately 8.5 g L^−1^) (Yilmaz and Göksungur 2024). Mechanistically, pH governs BC formation through at least three interconnected routes: (i) modulation of nutrient uptake efficiency, (ii) regulation of enzyme activities involved in cellulose precursor biosynthesis, and (iii) control of acidic by-product accumulation; deviation from the optimum amplifies acid stress and redirects carbon away from cellulose toward alternative metabolic pathways, reducing BC output (Montenegro-Silva et al. 2024).

Box–Behnken design-based RSM provided a statistically robust and predictive model (*R*^2^ = 0.95, Adj-*R*^2^ = 0.85, *F* = 9.96, *P* = 0.01), confirming that the three selected independent variables and their interactions accounted for the majority of variability in BC yield. Among these, glycerol concentration (X_1_) and inoculum size (X_2_) exerted significant effects in both linear and cross-interaction terms (*P* < 0.05), while incubation time (X_3_) contributed a weaker, non-significant effect. This hierarchy is physiologically consistent with the known primacy of carbon source availability and initial cell density in determining *Komagataeibacter* cellulose output, whereas moderate variation in culture duration, within the relatively narrow window tested, has less influence on final yield once carbon and inoculum conditions are optimized (Yilmaz and Göksungur 2024; Park et al. 2003).

Canonical analysis revealed mixed-sign eigenvalues (λ_1_ = 0.63, λ_2_ = 0.43, λ_3_ = − 0.91), confirming a saddle-point stationary point, an outcome that has been documented in other RSM-based fermentation optimization studies (Myers 1976; Lenth 2009). Saddle points arise when the response surface curves upward in some variable directions and downward in others, precluding the identification of a unique interior maximum. Furthermore, the coded value of X_3_ at the stationary point (1.49) fell outside the design constraints, rendering canonical path traversal statistically unjustified. Ridge analysis was therefore applied as the methodologically appropriate alternative, identifying the maximum feasible response along the path of steepest ascent within the design space (Myers 1976). The experimental yield obtained at the ridge-recommended optimum (6.2 g /160 mL) agreed closely with the model prediction (7.1 g /160 mL), confirming a model validation accuracy of 88.3% and demonstrating the practical reliability of the ridge-guided optimization strategy.

The optimized BC yield of 6.2 g /160 mL (equivalent to 39 g L^−1^) represents a 10.4-fold enhancement over the pre-optimization baseline (0.6 g /160 mL) and substantially surpasses most reported waste-based BC systems. When benchmarked against comparable processes from previous reported studies (Table 7), the advantage of the present route is evident in both final titer and volumetric productivity: BC production from BWH using *Gluconobacter oxydans* MG2021 reached 12.5 g L^−1^ after 14 days, while *K. nataicola* TISTR 2661 produced 13.9 g L^−1^ from banana peel after 9 days (Table 7). Expressed as average volumetric productivity, the present process delivers approximately 6.7 g L^−1^ day^−1^, compared with approximately 0.9 g L^−1^ day^−1^ for the bread waste acid hydrolysate system and approximately 1.5 g L^−1^ day^−1^ for the banana-peel system. Across the broader set of waste-derived static BC processes summarized in Table 7, which typically report titers of 4–15 g L^−1^ over 6–16 days from previous reported studies, the present hydrolysate-based process achieves a substantially superior combination of yield and production rate, establishing it as a high-performance benchmark for bread waste valorization into BC.

**Table 7:** Representative examples of BC production using laboratory -based medium and agro-industrial waste or by products-based medium

Producer	Production medium	Production time (days)	Yield (Dry weight) (g pellicle/L)	References
*Komagataeibacter sp*. strain HIJ12 EMCCN-4085	G. soli Bread waste hydrolysate-based medium	5.83	39 a	This study
*Komagataeibacter rhaeticus* UNIWA AAK2	Bread waste enzymatic hydrolysate-based medium	–	2.38	Pilafidis et al. (2025)
*Gluconobacter oxydans* MG2021	Bread waste acid hydrolysate-based medium	14	12.5b	Güzel (2024)
*Komagataeibacter rhaeticus* DSM 2004	enzymatic hydrolysates of stale bread-based medium	7	2.1	Esmail et al. (2025)
K*omagataeibacter rhaeticus* DSM 2004	Waste apple pulp extract	7	3.38	Esmail et al. (2025)
*Komagataeibacter nataicola* TISTR 2661	Banana peel-based medium	9.0	13.85	Moukamnerd et al. (2020)
*Gluconacetobacter xylinus* strain ATCC 53524	Modified HS medium	4.0	3.75	Mikkelsen et al. (2009)
*Acetobacter lovaniensis HBB5*	HS medium	7.0	0.04	Poyrazoglu and Biyik (2011)
*Acetobacter xylinum 0416*	HS medium	5.0	0.3722	Zahan et al. (2015)
*Gluconacetobacter xylinus strain*	carob and haricot bean (CHb) medium	9.0	1.8	Bilgi et al. (2016)
*Gluconacetobacter hansenii*	HS medium	22	0.017	Hutchens et al. (2007)
*Gluconacetobacter xylinus*	Medium cashew apple juice with soybean molasses	7.0	4.5	Souza et al. (2020)
*Mixed Komagataeibacter rhaeticus M12: Komagataeibacter intermedius* 6-5 = 2: 1	Pear pulp medium	7.0	10.94	Ma et al. (2021)
*Komagataeibacter intermedius* V-05	Soybean molasses and ethanol	14	10.0	Gomes et al. (2021)
*Gluconacetobacter xylinus* NRRL B-42	Waste glycerol and corn steep liquor	14	10.0	Vazquez et al. (2012)
*Komagataeibacter medellinensis* NBRC 3288	Overripe banana-based medium	12	4.0	Roda et al. (2017)
*Komagataeibacter xylinus* CICC No. 10529	Citrus peel and pomace-based medium	8	5.7	Islam et al. (2017)
*Acetobacter xylinum* NBRC 13693	Orange juice-based medium	14	5.9	Li et al. (2015)
*Gluconacetobacter xylinus* NRRL B-42	Waste glycerol and corn steep liquor-based medium	14	10	Mohammadkazemi et al. (2015)
*Gluconacetobacter entanii,*	Pecan nutshell-based medium	28	2.82	Dórame-Miranda et al. (2019)
*Komagataeibacter rhaeticus QK23*	Asparagus peel waste	25	2.57	Rodríguez-Soto et al. (2024)
*Komagataeibacter sp. CCUG73629*	Corncob-based medium	10	14.1	Akintunde et al. (2022)
*Komagataeibacter sp. CCUG73630*	Corncob-based medium	10	3.5	Akintunde et al. (2022)
*Komagateibacter xylinus*	Grape pomace–potato (GP)-based medium	6.0	4.0	Cazón et al. (2022)
*Komagateibacter xylinus*	Agro-industrial flour nitrogen source derivatives-based media	7.0	0.6–3.4	Absharina et al. (2026)
*Komagataeibacter xylinus*	Mango pulp waste-based medium	16	6.32	García-Sánchez et al. (2020)
*Komagataeibacter rhaeticus QK23*	Hydrolyzed Agro-Industrial Waste from Arti-Chokes-based medium	14	1.57	Quiñones-Cerna et al. (2025)

a: 102% (g BC/g BW) & b: 25% (g BC/g BW)

Future investigations should prioritize scale-up validation in larger bioreactor configurations to assess process robustness and oxygen transfer dynamics beyond flask scale. Expanding the RSM design space for incubation time and exploring fed-batch or semi-continuous feeding strategies may further improve yield. Additionally, establishing a quantitative link between fermentation conditions, BC pore architecture, and application-specific performance metrics, particularly for drug delivery matrices and wound-healing scaffolds, would strengthen the translational value of the present optimized process and guide rational medium design for structurally tailored BC production.

The purity and structural integrity of BC were markedly affected by both the culture medium and the purification strategy. BC obtained from HS medium displayed a well-defined nanofibrillar architecture following NaOH purification, whereas the hydrolysate-based BC retained visible impurities, likely resulting from the heterogeneous composition of the fungal BWH that contains proteins, lipids, salts, and Maillard reaction products (Skiba et al. 2022).

To enhance purity, an additional ethanol-gradient washing step was incorporated. Ethanol precipitation is widely recognized as an effective method for separating macromolecules based on solubility differences. In protein purification, particularly antibody recovery from plasma or serum, cold ethanol selectively precipitates large protein molecules such as immunoglobulins while leaving smaller contaminants in solution (Tscheliessnig et al. 2014). Similarly, in this study, stepwise ethanol treatment likely facilitated the differential precipitation of residual proteins and lipids adsorbed on the BC surface, leading to the removal of low-molecular-weight impurities and improved fibril definition. This suggests that ethanol not only serves as a dehydrating and denaturing agent but also promotes the selective removal of non-cellulosic materials, thereby improving the morphological purity of BC produced from complex waste substrates.

SEM images at 25,000× magnification revealed that HS-based BC displayed a dense, uniform fibril network with small, regular pores (Fig. 4C), whereas hydrolysate-based BC exhibited a heterogeneous structure with irregular fibril diameters and large pores (Fig. 4D). These morphological variations correlated with crystallinity differences (Table 4 and Fig. 5); the crystallinity of the produced BC was strongly affected by the composition of the growth medium. BC synthesized in HS medium exhibited a higher crystallinity index (CrI, 69%), consistent with previous reports showing that defined glucose-based media support the formation of well-ordered crystalline fibrils due to a steady glucose and mineral supply. In contrast, hydrolysate-based BC showed a markedly lower CrI (54%) with reduced diffraction peak intensity and broadened reflections, similar to findings from studies using fruit or lignocellulosic hydrolysates as substrates. This reduction in crystallinity is attributed to the heterogeneous sugar composition and residual impurities in hydrolysates, which disrupt the orderly assembly of cellulose chains. Overall, these results confirm that culture medium composition directly governs cellulose microstructure, with defined HS medium promoting higher crystallinity and hydrolysate-based media yielding more amorphous, flexible BC suitable for specific applications (Raghavendran et al. 2020; Khattak et al. 2014; Molina-Ramírez et al. 2017; Abol-Fotouh et al. 2020).

Thermogravimetric analysis (Fig. S2) further revealed that the HS-based BC demonstrated greater thermal purity and stability, characteristics that make it better suited for applications requiring high thermal resistance, such as reinforcement in composite materials. In contrast, the hydrolysate-based BC, while less thermally stable, exhibited greater water retention and higher residual content, properties that may be advantageous for biomedical and biotechnological applications where porosity, moisture handling, and surface reactivity are more critical than thermal stability (Costa et al. 2017).

FTIR spectra (Fig. S3) confirmed the presence of characteristic cellulose absorption bands in both HS- and hydrolysate-based BC, indicating preservation of the cellulose backbone. However, subtle spectral differences indicate variations in structural order. The hydrolysate-based BC (red line) shows a broader O–H stretching band (~ 3300–3400 cm^−1^), suggesting weaker and more disordered hydrogen bonding compared to the sharper band observed for the HS-based BC. Additionally, the reduced intensity in the C–O stretching region (1200–1000 cm^−1^) and slight shifts in peak positions reflect minor perturbations in glycosidic linkages and molecular packing (Shezad et al. 2010). These spectral changes imply a relative decrease in crystallinity and increased amorphous content in the hydrolysate-based BC, likely due to the complex and heterogeneous composition of the hydrolysate medium interfering with regular cellulose chain assembly (Skiba et al. 2022; Abol-Fotouh et al. 2020). Overall, the data confirm that while both samples retain the essential chemical structure of BC, the hydrolysate-based BC possesses a less ordered microstructure, consistent with a more amorphous and flexible form.

Although the absolute tensile strength values (Table 5) were lower than those reported in optimized BC systems (Hsieh et al. 2008; Yamanaka et al. 1989), the relative differences underscore the strong influence of culture medium and post-treatment on mechanical performance. The relatively low tensile values reflect the dry-state testing of thick pellicles rather than optimized thin films and are consistent with unmodified BC benchmarks under similar conditions. Overall, HS-based BC offers greater structural robustness suitable for mechanical applications, whereas the more porous hydrolysate-based BC, despite its reduced strength, may be advantageous for adsorption-based or biomedical applications where flexibility and surface reactivity are prioritized over mechanical stability.

The hydrophilic properties of both HS-based BC and hydrolysate-based BC (Table 6) revealed that both membranes exhibited high moisture content typical of BC, reflecting its strong hydrophilic nature and extensive hydrogen-bonded water networks (Abol-Fotouh et al. 2020; Klemm et al. 2005). While their overall hydration properties were similar, the hydrolysate-based BC showed higher water retention and porosity, likely due to its looser fibril structure and greater availability of hydroxyl groups formed under the complex carbon and nutrient composition of the hydrolysate medium (Castro et al. 2011). The consistent rehydration ratio between both types indicates that the core nanofibrillar framework remained intact despite differences in carbon source (Molina-Ramírez et al. 2017). The higher porosity observed in hydrolysate-based BC further supports its potential for applications requiring enhanced fluid exchange or molecular interaction, including heavy metal adsorption, wound healing, tissue engineering, and drug delivery systems (Castro et al. 2011).

## Conclusion

This study successfully demonstrated that microbially generated *G. soli* BWH constitutes a high-performance, sustainable alternative to conventional HS medium for BC production by *Komagataeibacter* sp. strain HIJ12 EMCCN-4085. Sequential OVAT screening systematically defined the critical physicochemical determinants governing BC biosynthesis, establishing a nutritionally robust baseline medium that eliminated the need for exogenous nitrogen supplementation, a practical advantage attributable to the inherent nutritional richness of the microbially generated hydrolysate. Subsequent BBD-based RSM, reinforced by canonical and ridge analyses, precisely refined glycerol concentration, inoculum size, and incubation time, delivering an exceptional BC dry weight of 39 g L^−1^ within 5.8 days, a tenfold enhancement over the unoptimized baseline and a volumetric productivity of approximately 6.7 g L^−1^ day^−1^ that substantially surpasses most reported waste-based BC systems. Structural characterization revealed that hydrolysate-based BC possessed a distinctively large pore size, enhanced porosity (41 ± 1.2%), and reduced crystallinity index (54%) relative to HS-based BC, while fully preserving the characteristic cellulose I chemical structure, a combination of properties that broadens its suitability for drug delivery, wound healing, tissue engineering, and filtration applications where high porosity and surface accessibility are prioritized over mechanical rigidity. Collectively, these findings establish microbially generated BWH as a compelling circular bioeconomy-aligned feedstock that simultaneously maximizes BC productivity and tailors material architecture, offering a scalable, low-cost, and environmentally responsible framework for converting food waste into high-value biomaterials with defined structural and functional properties.

## Supplementary Information

Additional file1 (TIF 91 kb): Fig. S1. Screening the effect of two independent variables on the synthesis of BC by *Komagataeibacter* sp. strain HIJ 12 EMCCN4085 via OVAT approach. (A) effect of using different organic nitrogen sources, and (B) effect of using different inorganic nitrogen sources.

Additional file2 (TIFF 663 kb): Fig. S2. Thermogravimetric analysis (TGA) and differential scanning calorimetry (DSC) curves of (A) HS-based BC and (B) hydrolysate-based BC.

Additional file3 (JPG 149 kb): Fig. S3. FTIR spectra of BC produced. HS-based BC, black line and hydrolysate-based BC, red line).

## Data Availability

The bacterial strain was submitted to the public Egyptian Microbial Culture Collection Network (EMCCN) (http://www.emccn.eg.net/index.php/en/emcc) at the National Research Center in Cairo, Egypt, with the accession number EMCCN-4085. Furthermore, the 16S rRNA nucleotide sequence of the bacterial strain was deposited to GenBank with the accession number PQ287240.1 (https://www.ncbi.nlm.nih.gov/nuccore/2798787990).

## Declarations

### Generative AI and AI-assisted technologies in the manuscript preparation process

During the preparation of this work the author(s) used AI- based tools in order to help in English editing of the manuscript. After using this tool/service, the author(s) reviewed and edited the content as needed and take(s) full responsibility for the content of the published article.
