# Supplementary figures and images for: Biocircular economy-driven bacterial cellulose with large pore size: statistical optimization using Glutamicibacter soli bread waste hydrolysate
Source: Bioresour Bioprocess. 2026 Jul 27;13(1):107. doi: 10.1186/s40643-026-01098-1 (PMC13407821; doi:10.1186/s40643-026-01098-1)

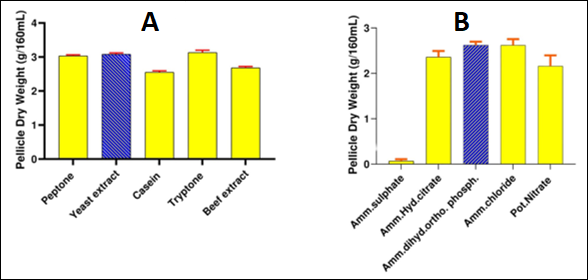

Supplement: Supplementary file 1 — Additional file1 (TIF 91 kb): Fig. S1. Screening the effect of two independent variables on the synthesis of BC by Komagataeibacter sp. strain HIJ 12 EMCCN4085 via OVAT approach. (A) effect of using different organic nitrogen sources, and (B) effect of using different inorganic nitrogen sources. [file 40643_2026_1098_MOESM1_ESM.tif]

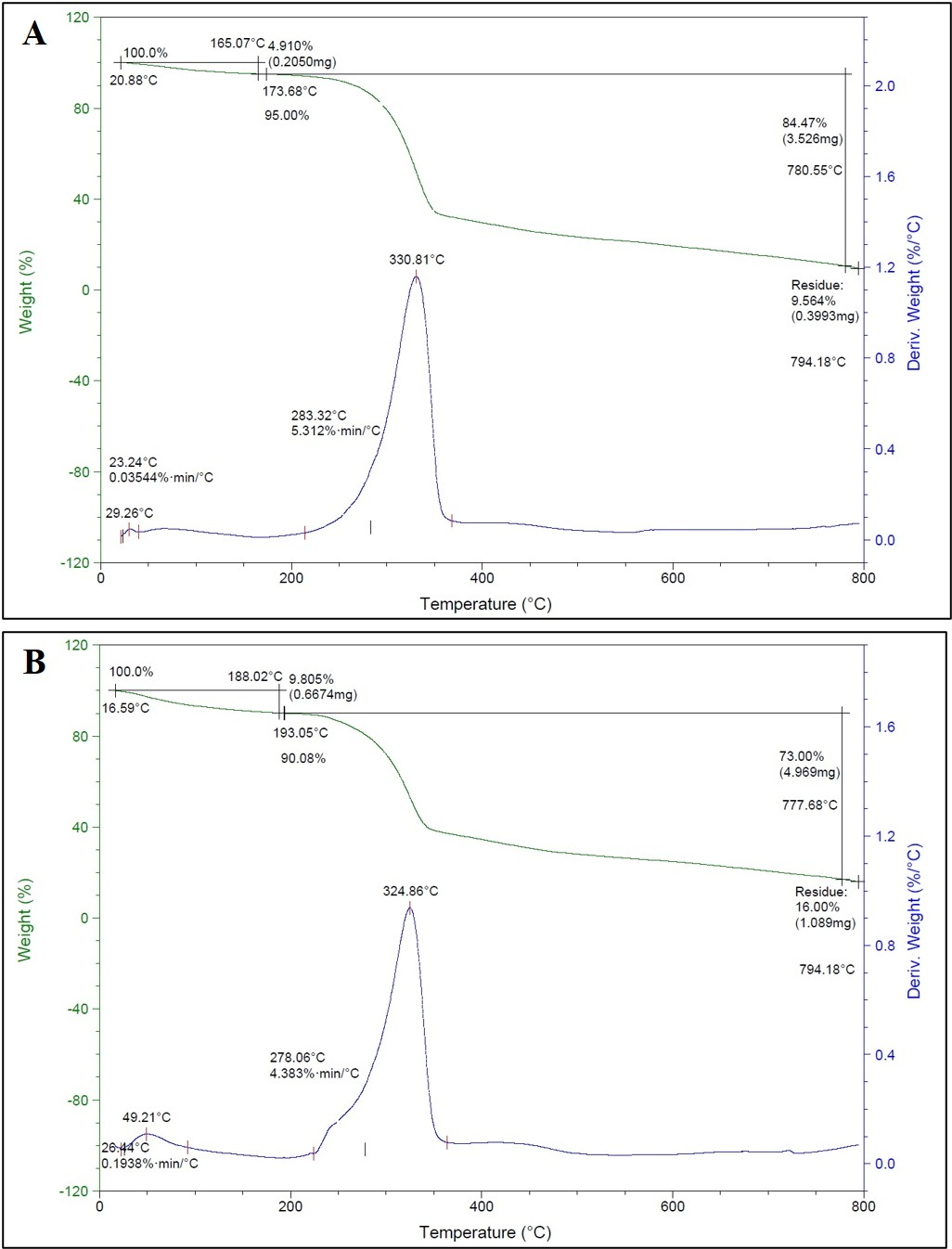

Supplement: Supplementary file 2 — Additional file2 (TIFF 663 kb): Fig. S2. Thermogravimetric analysis (TGA) and differential scanning calorimetry (DSC) curves of (A) HS-based BC and (B) hydrolysate-based BC. [file 40643_2026_1098_MOESM2_ESM.tiff]

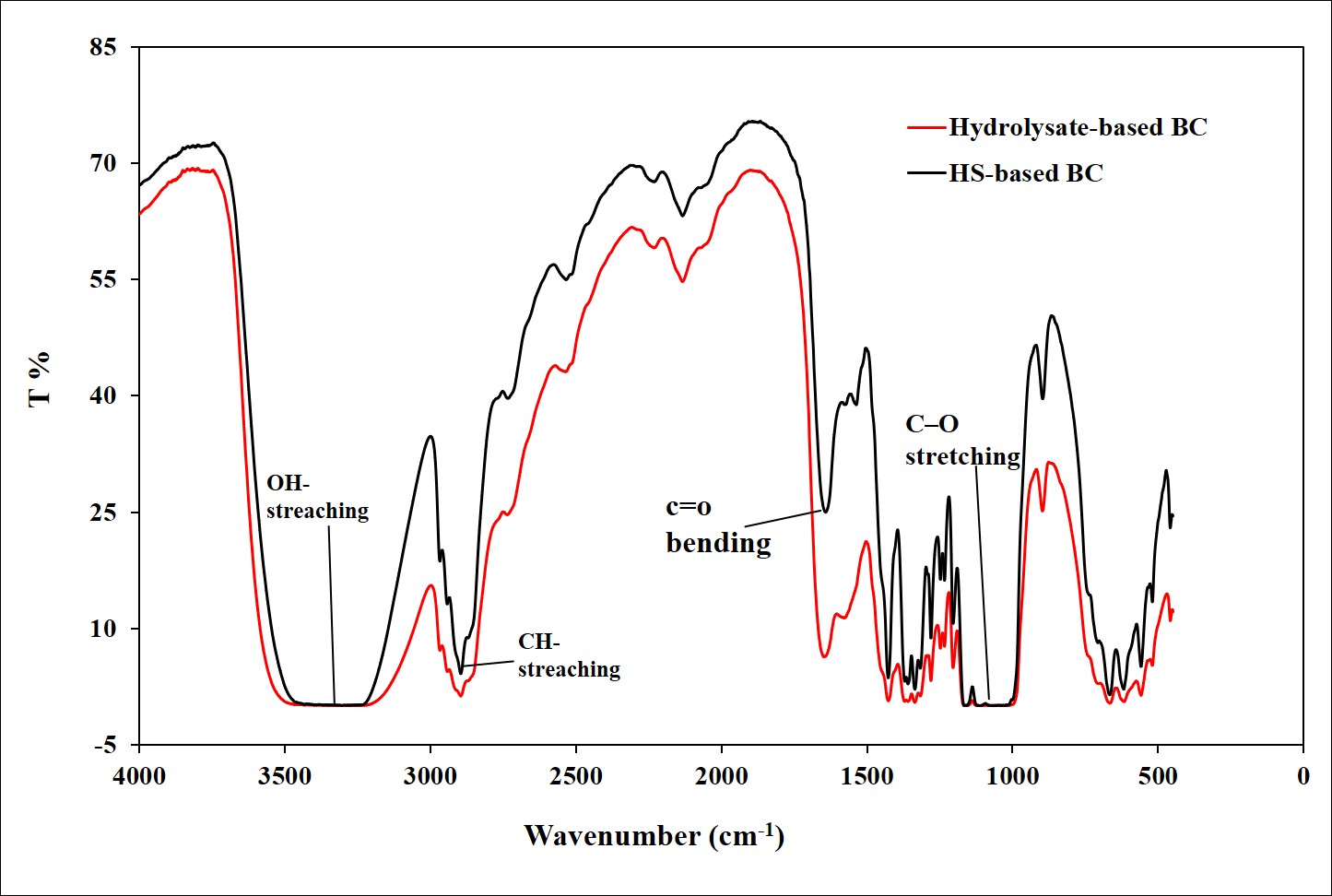

Supplement: Supplementary file 3 — Additional file3 (JPG 149 kb): Fig. S3. FTIR spectra of BC produced. HS-based BC, black line and hydrolysate-based BC, red line). [file 40643_2026_1098_MOESM3_ESM.jpg]
